# Can LLMs improve the accuracy of behavior prediction for personnel in high-stakes scenarios by predicting emotions? — A fine-tuning study based on scarce homogeneous cultural data in high-pressure environments

**DOI:** 10.1371/journal.pone.0352988

**Published:** 2026-07-08

**Authors:** Yibo Chen, Yang Ping, Shuhang Zhou, Caleb Jojo

**Affiliations:** 1 War Research Institute, Academy of Military Science of the People’s Liberation Army, Beijing, China; 2 College of Mechatronic Engineering, North University of China, Taiyuan, Shanxi, China; 3 McCormick School of Engineering & Applied Science, Northwestern University, Evanston, Illinois, United States of America; Teesside University, UNITED KINGDOM OF GREAT BRITAIN AND NORTHERN IRELAND

## Abstract

Research on human behavior prediction using LLMs is increasingly prevalent. This study addresses a key question in the interdisciplinary fields of behavioral science and artificial intelligence: Can LLMs enhance the accuracy of predicting personnel behavior by predicting emotions in high-pressure scenarios? We rigorously screened homogeneous cultural data for personnel in high-stakes roles, constructing the first multidimensional dataset of “scenario-emotion-behavior” under these conditions. Following the fine-tuning of the LLM based on this dataset, we evaluated its behavior prediction accuracy. Experimental results reveal that emotion-predicting LLMs outperform baseline LLMs and behavior-predicting LLMs on multiple metrics, emphasizing the crucial roles of the Emotion-Imbued Choice Model and Behavioral Decision Theory in enhancing LLM behavior prediction capabilities. This study promotes the interdisciplinary integration of AI with cognitive and behavioral science, offering fresh insights into high-risk behavior domains and establishing a novel paradigm where LLMs improve behavior prediction accuracy through emotion prediction.

## Introduction

In high-risk environments saturated with highly adversarial, time-sensitive conditions, accurately predicting personnel actions is critical for outcomes, safety, and operational success. Recent advancements in NLP (Natural Language Processing) technologies and LLMs have fueled research in enhancing human behavior prediction capabilities. Notably, behavioral decisions in such contexts are influenced by emotional responses. Studies confirm that emotional and behavioral patterns are regular in high-pressure scenarios [1,2]. Behavioral theories like the “Emotion-Imbued Choice Model” and “Behavioral Decision Theory” elucidate the interplay between emotions and decisions in uncertain conditions. The widespread application of these theories in various contexts reveals how scenarios trigger emotions, how emotions influence behavioral decisions, and the characteristic irrational preferences in human behavior [3–7]. While machine learning methods for analyzing human behavior patterns, such as BEAST [8] and LLM agents utilizing simulated biosensor signals like EduAgent [6], demonstrate that neural network models can learn intrinsic human behavioral patterns, these studies fail to consider the potential impact of emotions.

Collectively, these studies point to a critical scientific question: Can LLMs improve the accuracy of behavior prediction for personnel in high-stakes scenarios by predicting emotions?

Multidimensional datasets that specifically depict “scenario-emotion-behavior” of personnel in real high-pressure situations are extremely rare. This scarcity results from the sensitivity of such data and the challenges associated with simultaneously annotating emotions and behaviors. To address this issue, we collected a considerable amount of publicly available video material, carefully designed an annotation framework, and selected high-quality samples to create a “scenario-emotion-behavior” dataset for LLM fine-tuning. Importantly, we made a concerted effort to control our data sources to include personnel from homogeneous cultural backgrounds, thereby minimizing the impact of cultural differences and diverse organizational systems on emotional responses and behavioral patterns [9].

This research hypothesizes that LLMs can learn homogeneous cultural behavior patterns through fine-tuning and that integrating Behavioral Decision Theory can bolster behavior prediction effectiveness. The main challenge is the scarcity of suitable training data. Traditional datasets focus either on emotional responses or behavioral decisions, often neglecting the multifaceted relationship between scenario context, emotion, and decision-making. To address this gap, we compiled a large corpus of publicly available video materials and established a systematic annotation framework to create a “scenario-emotion-behavior” dataset [10–13].

**Fig 1:**
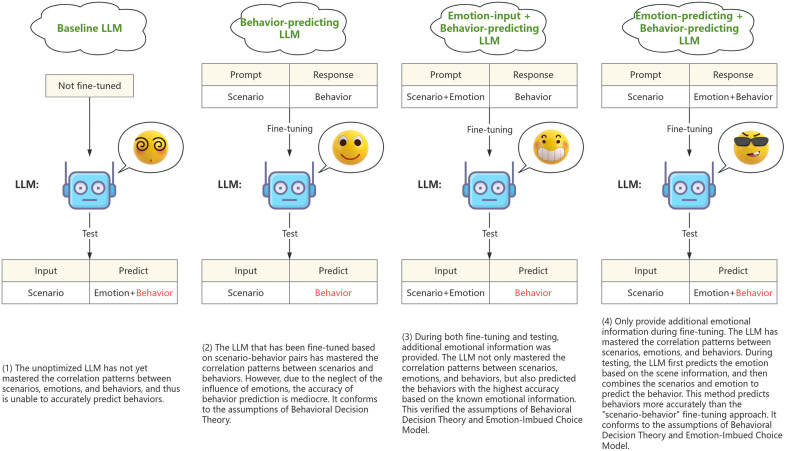
Optimization paths comparison. There are a total of four optimization paths, each optimization path involves different inputs and outputs, and the fine-tuning format and testing format for each optimization path remain consistent.

As shown in “Fig 1”, based on the aforementioned multidimensional dataset, we systematically fine-tuned the LLM and compared different optimization paths: (1) baseline LLM; (2) behavior-predicting LLM; (3) emotion-input + behavior-predicting LLM; (4) emotion-predicting + behavior-predicting LLM. Experimental results demonstrate that the LLM performing emotion prediction outperformed both the baseline LLM and the behavior-predicting LLM in behavior prediction tasks on multiple metrics, strongly validating our hypothesis.

The main work and contributions of this study:

1. Proposing a systematic method for constructing a scenario-emotion-behavior dataset for personnel in high-stakes roles. Using homogeneous cultural samples, we established a multidimensional annotation system integrating scenario inputs, emotion annotations, and behavioral decision annotations, and provided a high-quality instance of a scenario-emotion-behavior dataset. To our knowledge, this represents one of the early comprehensive efforts in this field, offering a replicable methodological framework for constructing similar multidimensional datasets in high-risk environments in the future.
2. This study verifies that LLMs fine-tuned with data empowered by Behavioral Decision Theory can to some extent enhance their accuracy in predicting personnel behavior in high-pressure scenarios, confirming that LLMs can, to some extent, learn the intrinsic patterns of homogeneous cultural behavior through fine-tuning.
3. To our knowledge, this study is the first to validate that LLMs, fine-tuned with data jointly empowered by the Emotion-Imbued Choice Model and Behavioral Decision Theory, can further improve their accuracy in predicting personnel behavior in high-pressure scenarios, by predicting immediate emotions of individuals. It initially reveals the crucial bridging role of emotional information in enhancing LLMs’ behavior prediction capabilities in critical contexts, establishing a novel optimization paradigm where LLMs improve behavior prediction accuracy by predicting emotions.

## Behavioral theory empowerment

### Basic emotions theory

The Basic Emotions Theory [14], proposed by psychologist Paul Ekman, posits that humans possess a set of universal, cross-cultural basic emotions. These emotions are biologically driven and typically manifest through specific facial expressions and physiological responses. Ekman initially identified six basic emotions: happiness, anger, fear, sadness, surprise, and disgust. Subsequent studies [15] expanded this theory, enabling the categorization of compound emotions formed from combinations of these basic emotions. In our analysis, we have utilized this theory to describe various emotions experienced by individuals accurately by using single-word descriptors. To achieve standardized annotation procedures and training goals, we only use one emotion word in each annotation. We considered that if multiple emotion words were used for emotion description, it would not only lead to an exponential increase in the demand for the size of the training set, but also the overlapping semantics or emotions among multiple emotion words might cause unexpected information redundancy for LLM training, resulting in errors. Although using a single compound emotion word might result in the loss of a small amount of emotional information, based on the current behavioral research foundation, it is the most applicable solution, and this study will verify the effectiveness of this method. Currently, most datasets and corpora are single-label annotated based on basic emotions [16], how to truly accurately record emotion information in words remains an unsolved topic in the scientific community, and therefore this might have limited the upper limit of the accuracy of this study. Overall, the current method enables a standardized classification of compound emotions for effective data analysis.

### Behavioral decision theory

Behavioral Decision Theory [17] investigates how individuals make decisions within complex and uncertain environments, focusing on the influence of cognitive biases and situational factors on decision-making. Unlike traditional rational decision-making models, Behavioral Decision Theory asserts that individual decisions are frequently swayed by irrational factors, which makes traditional rule-based methods for predicting behavior ineffective. Numerous studies have successfully trained decision-making agents using datasets informed by Behavioral Decision Theory, yielding significant outcomes [6,7]. It can be seen that Behavioral Decision Theory emphasizes the correlation between scenarios and behavioral decisions. The above research indicates that LLMs fine-tuned based on scenario-behavior datasets can acquire to some extent the decision-making patterns of “incomplete rationality” of humans, thereby verifying that Behavioral Decision Theory can be applied to the fine-tuning of LLMs for behavioral prediction. Based on this, this study incorporated the second optimization path, behavior-predicting LLM.

Furthermore, the Behavioral Decision Theory posits that decision-making should be regarded as a cultural phenomenon. The influence of culture on decision-making can be seen as a kind of social pressure that unintentionally or intentionally forces decision-makers to perceive, decide, and act in accordance with their own cultural traditions. This indicates that the more a scenario-behavior dataset comes from a homogeneous culture, the more it can represent the behavioral patterns within that cultural background [9]. This determines that this study adopts the strategy of collecting data from homogeneous cultures.

### Emotion-imbued choice model

The Emotion-Imbued Choice Model [18] emphasizes the significant role that emotions play in individual decision-making processes. This model highlights how emotions influence cognitive evaluations and behavioral choices. It posits that emotions are not merely reactions to external stimuli; rather, they serve a regulatory function during decision-making. This is particularly pertinent in scenarios characterized by uncertainty or high risk, where emotions can heavily influence individual choices. Recent studies [3–5] have highlighted the significant impact of emotional cognition on decision-making agents.

Based on Behavioral Decision Theory and Emotion-Imbued Choice Model, this study holds that if the LLM used for behavior prediction incorporates both scenario and emotion factors during fine-tuning, and also provides scenario information and emotion information when making predictions, its prediction effect will be the best among all the LLM fine-tuning methods in this paper. Therefore, this study proposes the third optimization path, emotion-input + behavior-predicting LLM. However, in practical applications, usually only specific scenario are known, while emotion information is difficult to obtain. Therefore, this study believes that if the LLM used for behavior prediction incorporates both scenario and emotion factors during fine-tuning, and only provides scenario information when making predictions, requiring it to complete the missing emotions by itself, its behavior prediction effect will be second only to emotion-input + behavior-predicting LLM. This is the fourth optimization path proposed by this study, emotion-predicting + behavior-predicting LLM.

## Dataset construction

**Fig 2:**
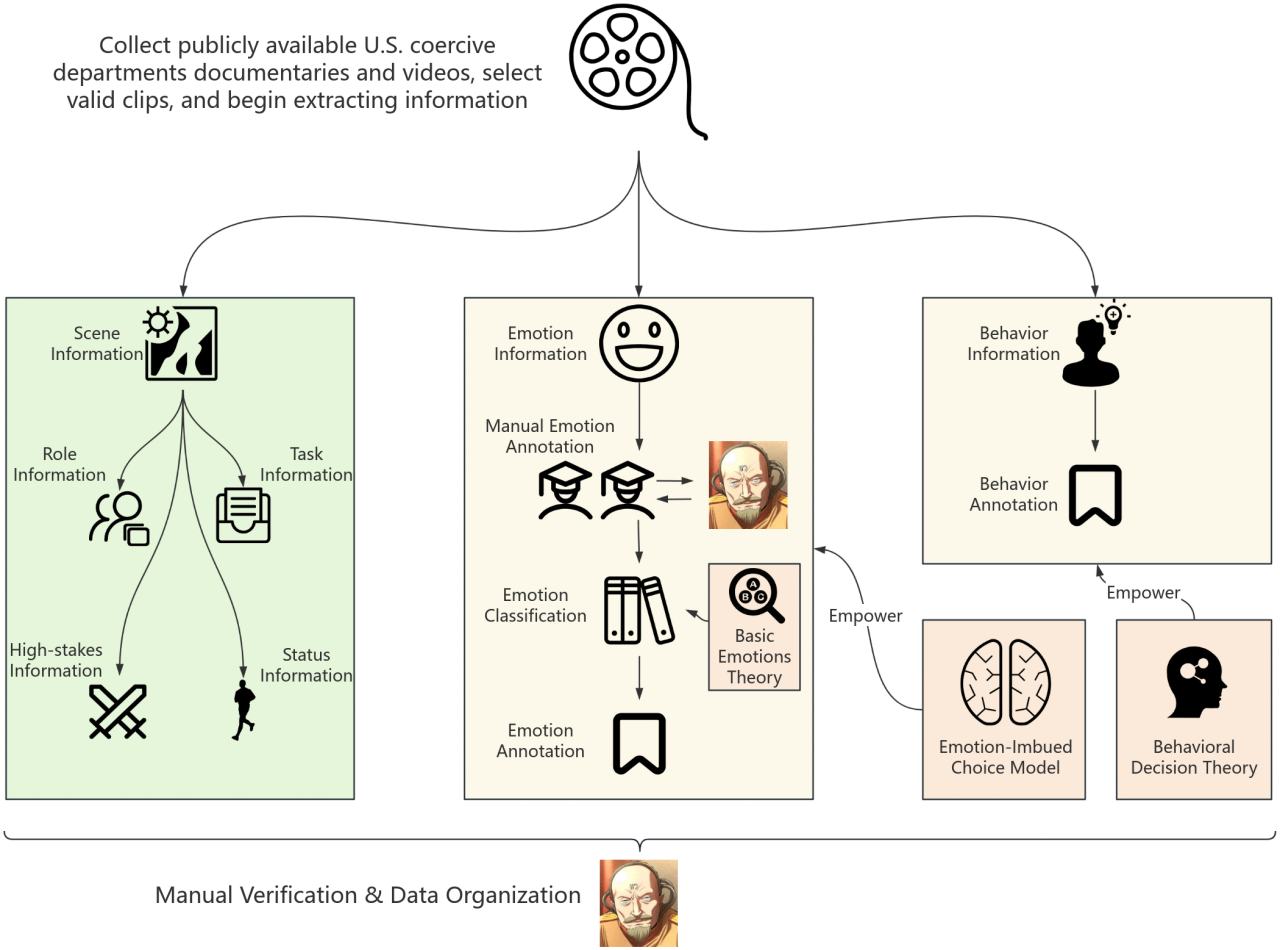
Dataset construction methodology. A multidimensional annotation system to describe four aspects of each scenario: “Role Information,” “Task Information,” “Context Information,” and “Status Information.” Each sample underwent emotion and behavior decision annotations led by a psychology officer and two computer science graduate students, with rigorous measures for objectivity and accuracy.

We believe that LLMs can learn homogeneous cultural behavior patterns through fine-tuning, enhancing prediction effectiveness by integrating Behavioral Decision Theory. The primary challenge lies in data scarcity; traditional datasets typically focus on emotional responses or behavioral decisions, often overlooking their intricate relationships. Our comprehensive annotation framework represents a pioneering approach to creating a “scenario-emotion-behavior” dataset, filling existing gaps in the literature. To scientifically construct a “scenario-emotion-behavior” dataset for LLM fine-tuning, we designed a series of explicit steps. “Fig 2” illustrates the process from data collection to annotation completion in this study.

### Data sources

We selected U.S. law enforcement and security agencies as the primary data source due to the availability of public video materials depicting high-stakes operations. To ensure cultural homogeneity, we restricted our data to documentaries and videos featuring personnel from these U.S. departments. From publicly available documentaries and videos, we rigorously screened samples meeting all the following criteria: clearly describing specific operations or missions in which the personnel participated, with specific high-pressure scenarios; clearly and specifically displayed emotions, with visible facial expressions capable of reflecting the psychological state of individuals in specific scenarios; clearly demonstrated behavioral decisions of individuals, allowing the extraction of behavioral decisions from language and actions. Samples cover diverse scenarios including various challenging environments such as urban patrols, emergency response, and public safety operations. The temporal span of the data ranged from the mid-20th century to the present (approximately 1950s to 2020s), ensuring the content originates entirely from real modern high-stakes scenarios. We ensured that the samples collected had an appropriate proportion of positive cases, negative cases, official records, and unofficial records, to ensure the diversity of the scenarios. In a few controversial scenes, we cross-referenced information from multiple sources to verify the objective situation, and endeavored to avoid editorial bias and selection errors.

### Annotation framework

We developed a multidimensional annotation system to describe four aspects of each scenario: “Role Information,” “Task Information,” “Context Information,” and “Status Information.” “Role Information” refers to the departmental unit and functional role of the personnel (e.g., “Patrol officer in a city police department”). “Task Information” refers to the task currently being executed by the personnel’s unit at the given moment (e.g., “Responding to a public disturbance call”). “Context Information” refers to the specific situational details known to the personnel at the current moment (e.g., “The situation involves a potentially armed individual in a crowded area; backup units are en route”). “Status Information” refers to the specific behavioral state of the personnel at the current moment (e.g., “Currently positioned behind vehicle cover, communicating with dispatch”). These four parts comprehensively describe the scenario information known to the specified individual at the given moment.

Each sample underwent emotion and behavior decision annotations led by a psychology officer and two computer science graduate students, with rigorous measures for objectivity and accuracy:

- Emotion Annotation: Based on video clips and manual descriptions, emotions were described manually. The emotion of the specified individual at the current moment in each clip was accurately expressed using a single word in the annotator’s native language. Due to the subjective nature of emotion annotation, this study was completed collaboratively by three staff members (including a single psychology officer and two computer science graduate students) to ensure objectivity and accuracy as much as possible. The three staff members were divided into one two-person team and one single-person team. The two-person team (including two computer science graduate students) served as annotators, each describing the emotion using a single word. The single-person team (which included a single psychology officer) served as the arbitrator, responsible for adjudicating disagreements arising within the two-person team. When the two-person team’s emotion descriptions showed no discrepancy, the description was considered complete. When discrepancies arose in the descriptions within the two-person team, an internal discussion was held first. If a consensus was reached, the description was considered complete. If consensus could not be reached, a disagreement occurred, and the single-person team adjudicated the most suitable description. After completing the emotion description, the emotion description for each sample was classified using a cross-language emotion dictionary [19], and the emotion annotation was determined according to compound emotion categories. In this study, the Cohen’s Kappa coefficient for emotion categories between the two-person team was 0.71.
- Behavior Decision Annotation: The specific compound behaviors of the individuals in the video clips were annotated (e.g., “Maintain cover and request additional support”). The specific content of the behavior decision annotations was developed by two graduate students in computer science through discussion. It mainly describes in detail from four aspects shown by the main character in the video: mobility, using tools (performing operations), avoiding hazards, and communicating with colleagues. If a certain aspect of behavior is not exhibited in the video, that aspect will not be described. Unlike the emotion annotations, the behavior labels were produced through a consensus-based workshop procedure rather than independent pre-discussion coding, because the behavior descriptions were open-ended, compound, and multi-dimensional. Two annotators jointly analyzed and discussed each sample based on predefined decision rules before generating the final behavior label. For the very few samples where consensus could not be reached, the final annotation was determined by the psychology officer during the confirmation phase. Finally, these annotations were confirmed by a psychology officer. The behavior labels should be regarded as consensus-based annotations grounded in explicit coding rules, rather than independently coded annotations suitable for estimating standard inter-annotator reliability.

All annotated samples underwent secondary verification to ensure the consistency and accuracy of the scenario information, emotion annotation, and behavior annotation. The dataset was organized into pure text data, and no personally identifiable, sensitive, or private information was collected. Manual verifiers checked based on the video clips and original footage to ensure each sample matched the actual descriptions in the documentaries or videos. After verification, ambiguous or incorrect annotations were corrected. Additionally, samples that were completely identical in content were deleted.

### Ethical approval

This study is a conducting research using legally obtained public data. It did not involve:

- Any direct interaction with human or animal subjects.
- The collection of personally identifiable, sensitive, or private information from any source.
- Any intervention in the physical, psychological, or social integrity of individuals.

According to China *Measures for Ethical Review of Life Sciences and Medical Research Involving Human Beings (2023)*, research using legally obtained public data or data acquired through observation of public behavior without interference is typically exempt from requiring formal approval by an Institutional Review Board (IRB) or an Ethics Committee. The Ethics Committee of the Academy of Military Science has confirmed that this study does not require formal ethical approval. During the data recording process, any information containing potentially identifiable characteristics such as specific times, locations, personal traits, and event names, was avoided.

## Validation experiments

### Optimization paths

We fine-tuned various LLM architectures using the “scenario-emotion-behavior” dataset, including ERNIE-4.0-Turbo-8K, Meta-Llama-3.1-8B, Qwen2.5-7B-Instruct, GPT-3.5-turbo-1106, and ChatGLM3-6b. We implemented a LoRA fine-tuning method, using the AdamW optimizer and measuring performance with metrics such as BERTScore and ROUGE.

We evaluated the LLMs through ablation experiments across four optimization paths:

1. Baseline LLM (unoptimized).
2. Behavior-predicting LLM (based on Behavioral Decision Theory).
3. Emotion-input + behavior-predicting LLM (based on Behavioral Decision Theory and Emotion-Imbued Choice Model, incorporating emotion descriptions).
4. Emotion-predicting + behavior-predicting LLM (based on Behavioral Decision Theory and Emotion-Imbued Choice Model, emotions are obtained through prediction).

By comparing the baseline LLM and the behavior-predicting LLM, we can verify whether LLMs can learn the intrinsic patterns of homogeneous cultural behavior to some extent through fine-tuning; by comparing the behavior-predicting LLM and the emotion-predicting + behavior-predicting LLM, we can verify the effect of emotion prediction in enhancing LLMs’ behavior prediction capability; by comparing the emotion-predicting + behavior-predicting LLM and the emotion-input + behavior-predicting LLM, we obtain the improvement effect on behavior prediction when emotions are given as known conditions, which can further validate the data empowerment effect of the Emotion-Imbued Choice Model and the potential role emotions may play in LLM behavior prediction. In this study, the Emotion-input + behavior-predicting LLM demonstrated the upper bound of the Emotion-Imbued Choice Model fine-tuning method. Unless we can simultaneously obtain both scenario information and emotion information, we cannot use it as a directly deployable system. This is why the Emotion-predicting + behavior-predicting LLM was proposed, to achieve further improvement solely based on scenario information. ERNIE-4.0-Turbo-8K is a representative advanced model among current foundation LLMs, making it highly suitable for fine-tuning research. To establish an objective and comparable evaluation benchmark, we selected it as the baseline model for the ablation experiments.

### Prompt template

We instruct the LLM to first predict emotions, and then predict behaviors based on the scenario and emotions. Due to the LLM’s autoregressive capability, it can “see” the emotions it has already predicted while generating behavioral predictions. We do not split this into two separate prompts; instead, we adopt a zero-shot single prompt. This allows the LLM to operate independently, thereby avoiding overly complex calls while achieving the effect of controlling experimental variables.

Behavior-predicting LLM prompt template: *Your current role is XXX. Your current mission is XXX. You learn that XXX. You are currently XXX. Please state your behavioral decision based on the current situation, no more than 50 words.*

Emotion-input + behavior-predicting LLM prompt template: *Your current role is XXX. Your current mission is XXX. You learn that XXX. You are currently XXX. Your current emotional state is XXX. Based on the current situation and your emotions, please state your behavioral decision, no more than 50 words.*

Emotion-predicting + behavior-predicting LLM prompt template: *Your current role is XXX. Your current mission is XXX. You learn that XXX. You are currently XXX. Please state your current emotion and based on the current situation and your emotion, provide your behavioral decision. Response in the format “Emotion; Behavioral Decision,” no more than 50 words.*

### Dataset split

To ensure scientific and reliable model fine-tuning and testing processes, we randomly splited the dataset into training, validation, and test sets at an 80%/10%/10% ratio based on individuals. The division of individuals is based on the recording of the same individual from the same video during the dataset construction process. Prior to model evaluation, to exclude test set samples that were overly similar to the training set, we removed samples with a cosine similarity exceeding 0.85 (4 samples were removed). The training set was used for model fine-tuning, the validation set for periodically evaluating model performance during training to facilitate hyperparameter tuning, model selection, or early stopping, thereby preventing overfitting and improving generalization capability; the test set was used for assessing model accuracy, excluded from LLM fine-tuning, and employed solely during model evaluation. This approach mitigates the risk of data leakage from related clips across different splits, ensuring that each model is evaluated on distinct individuals and reducing the likelihood that similar language or situational context influences performance during testing. The test set incorporates diversified scenarios, and it contains 100 samples.

### Model fine-tuning training

During the model fine-tuning phase, we employed the LoRA fine-tuning method [20], updating only partial parameters during fine-tuning, with LoRARank set to 64 and LoRAAlpha = LoRARank. First, initialization settings were applied to the selected LLM, loading pre-trained weights to enhance training efficiency [21]. Subsequently, the AdamW optimizer [22] was adopted for parameter optimization, with a learning rate of 1e-6, employing a linear learning rate decay strategy to gradually reduce the learning rate for more stable convergence [23]. The Cross-Entropy Loss function was selected to measure the error between model outputs and target annotations [24]. The random seed is set to 42.

During fine-tuning, we conducted iterative training using the training set with a batch size of 16, evaluated model performance on the validation set after each training epoch, and set the maximum training epochs to 5. Additionally, mixed-precision computation [25] was utilized to improve fine-tuning efficiency. The early stopping criterion is set to Validation Loss being less than 0.01, and the Early Stopping Patience is set to 1.

The hardware configuration used for the fine-tuning was the Kunlun Core P800 cluster.

### Evaluation metrics and baseline settings

The performance of fine-tuned models was evaluated through the following metrics [26]: (1) BERTScore F1 [27], measuring semantic alignment between generated and reference texts; (2) ROUGE series metrics [28], including ROUGE-1, ROUGE-2, and ROUGE-L, quantifying similarity between generated and reference texts; (3) Satisfaction score, a simple expert rubric, as a supplementary assessment grounded in domain expertise, aligned with the action selection criteria—encompassing positional awareness, tool usage, strategic actions, and collaborative communication—of mainstream simulation agents [29], percentage-based normalization to quantify the prediction accuracy of the LLM. Specifically, we hired four experts in the field of artificial intelligence. Each expert was assigned one of the following evaluation tasks: Does the result set match the consistency with the reference set regarding whether to make a maneuver (i.e., whether to change position)? Does the result set match the consistency with the reference set regarding whether to use tools/handle hazards (i.e., whether to take active actions)? Does the result set match the consistency with the reference set regarding whether to hide/dodge hazards (i.e., whether to take passive actions)? Does the result set match the consistency with the reference set regarding whether to communicate with colleagues to obtain/support help and assistance? Each expert gave an integer score out of 10, with 10 indicating almost complete consistency and 0 indicating almost complete inconsistency. The model conditions are kept confidential from the raters. Finally, each result set received a normalized average as the satisfaction score.

We present the results of each indicator in the form of an average value and a 95% confidence interval, and explain the significance test results in text form in the corresponding sections.

### Generalizability testing

To validate the generalizability of the emotion-predicting + behavior-predicting LLM optimization approach across different LLMs, we fine-tuned multiple LLMs based on the “scenario-emotion-behavior” dataset. The baseline and experimental results for each LLM are shown in “Table 1”.

**Table 1:** Different LLMs and their fine-tuning evaluation results.

Model	BERTScore	ROUGE-1	ROUGE-2	ROUGE-L	Satisfaction Score
**ERNIE-4.0**	65.96% [65.13%, 66.78%]	17.99% [16.16%, 19.82%]	4.00% [2.54%, 5.46%]	16.93% [15.29%, 18.57%]	62.5% [54.5%, 70.5%]
**Fine-tuned ERNIE-4.0**	73.32%* [71.67%, 74.96%]	38.51%* [34.33%, 42.68%]	16.06%* [11.65%, 20.47%]	35.95%* [31.90%, 40.00%]	75.0%* [65.8%, 84.2%]
**Llama-3.1**	63.63% [62.85%, 64.40%]	11.24% [9.79%, 12.70%]	2.27% [1.45%, 3.09%]	9.31% [8.07%, 10.54%]	55.0% [45.8%, 64.2%]
**Fine-tuned Llama-3.1**	73.01%* [71.74%, 74.27%]	38.21%* [34.71%, 41.72%]	16.65%* [11.90%, 21.39%]	36.82%* [33.36%, 40.27%]	80.0%* [80.0%, 80.0%]
**ChatGLM3**	61.69% [60.99%, 62.38%]	8.16% [7.11%, 9.20%]	0.87% [0.30%, 1.45%]	6.80% [5.91%, 7.68%]	62.5% [54.5%, 70.5%]
**Fine-tuned ChatGLM**	64.43%* [63.53%, 65.34%]	13.84%* [12.50%, 15.18%]	2.30%* [1.57%, 3.03%]	12.61%* [11.40%, 13.83%]	72.5%* [64.5%, 80.5%]
**GPT-3.5**	67.51% [66.70%, 68.32%]	16.13% [14.58%, 17.68%]	6.13% [3.36%, 8.90%]	14.44% [13.10%, 15.78%]	47.5% [39.5%, 55.5%]
**Fine-tuned GPT-3.5**	69.48%* [68.01%, 70.95%]	26.69%* [23.69%, 29.68%]	10.59%* [7.89%, 13.30%]	25.60%* [22.71%, 28.50%]	62.5%* [54.5%, 70.5%]
**Qwen2.5**	62.15% [61.41%, 62.89%]	10.11% [9.20%, 11.03%]	1.01% [0.45%, 1.57%]	8.92% [8.16%, 9.69%]	60.0% [60.0%, 60.0%]
**Fine-tuned Qwen2.5**	73.59%* [72.17%, 74.95%]	38.46%* [34.30%, 42.62%]	19.61%* [14.05%, 25.17%]	36.31%* [32.31%, 40.30%]	72.5%* [64.5%, 80.5%]

The test results of each LLM and its fine-tuned model. The results of each indicator are presented in the form of average values and 95% confidence intervals (in brackets).

* Denotes statistical significance (p-value<0.05) compared to the un-tuned model.

It can be observed that the behavior prediction capabilities of various fine-tuned LLMs improved, with satisfaction scores increasing by 10.0–25.0 percentage points, and the highest satisfaction score reached 80.0%. Both BERTScore and ROUGE series metrics also improved, with BERTScore increasing by 1.97–11.44 percentage points, indicating that the emotion-predicting + behavior-predicting LLM optimization approach is effective and exhibits certain generalizability. All metrics still retain room for improvement. If the dataset sample size is further expanded and tailored, fine-tuning methods optimized for each distinct LLM architecture are applied, and the performance may be enhanced.

We conducted paired t-tests for each metric of each fine-tuned LLM and their base LLMs. All comparisons showed statistical significance (p < 0.05). Exact p-values and mean differences along with their 95% confidence intervals, are presented in Table 3 in the S1 Appendix. Multiple comparisons are present, so the significance levels should be interpreted with caution.

**Fig 3:**
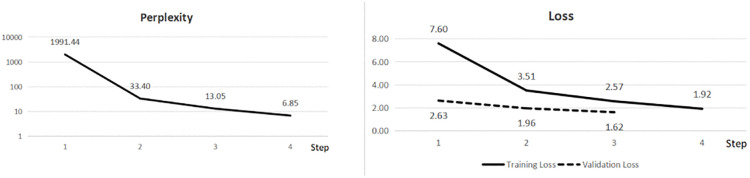
Fine-tuning process. Perplexity, Training Loss, and Validation Loss all decreased gradually at a decelerating rate.

Taking ERNIE-4.0-Turbo-8K as an example during fine-tuning, post-fine-tuning testing on the training set yielded a BERTScore of 74.52%, ROUGE-1 of 37.03%, ROUGE-2 of 17.87%, and ROUGE-L of 36.59%, showing minimal difference from the test set results. As shown in “Fig 3”, Perplexity, Training Loss, and Validation Loss all decreased gradually at a decelerating rate, indicating effective model learning without signs of overfitting.

### Emotion annotation ablation experiment

In the generalization experiments, we have already tested the capabilities of the baseline LLM and the emotion-predicting + behavior-predicting LLM. In the ablation experiments, we respectively treated emotion information as an irrelevant variable or as input to test, thereby comparing the incremental effect brought by emotion information.

We conducted two additional ablation experiments. In the first ablation experiment (behavior prediction test without emotion), we adopted the behavior-predicting LLM optimization approach, with fine-tuning data empowered by Behavioral Decision Theory, using only scenario descriptions + behavior annotations for fine-tuning, meaning all emotion annotations were removed from the dataset (including the test set). In the second ablation experiment (behavior prediction test with emotion input), we adopted the emotion-input + behavior-predicting LLM optimization approach, with fine-tuning data jointly empowered by the Emotion-Imbued Choice Model and Behavioral Decision Theory, using scenario and emotion descriptions + behavior annotations for fine-tuning, meaning all emotion annotations originally in the output of the dataset (including the test set) were moved to the input, so the input information included both scenario and emotion information. The results of different optimization paths compared to the baseline are shown in “Table 2”.

**Table 2:** Ablation experiment evaluation results (Model: ERNIE-4.0-Turbo-8K).

Configuration	BERTScore	ROUGE-1	ROUGE-2	ROUGE-L	Satisfaction Score
**Base Model**	64.05% [62.98%, 65.11%]	12.26% [10.00%, 14.53%]	3.06% [1.77%, 4.36%]	11.31% [9.28%, 13.35%]	62.5% [54.5%, 70.5%]
**FT Model**	68.97%* [66.90%, 71.04%]	24.43%* [19.31%, 29.54%]	6.18%* [3.82%, 8.53%]	20.98%* [16.47%, 25.49%]	75.0%* [65.8%, 84.2%]
**w/o Emo. Model**	63.74% [62.72%, 64.76%]	11.45% [9.38%, 13.52%]	1.37% [0.54%, 2.21%]	8.97% [7.42%, 10.51%]	57.5% [49.5%, 65.5%]
**w/o Emo. FT Model**	67.31%* [65.98%, 68.65%]	18.97%* [15.98%, 21.95%]	0.95% [0.62%, 1.28%]	16.13%* [13.54%, 18.71%]	67.5%* [59.5%, 75.5%]
**Emo. input Model**	65.76% [64.72%, 66.81%]	18.34% [15.15%, 21.53%]	3.93% [1.57%, 6.29%]	14.14% [11.79%, 16.50%]	62.5% [54.5%, 70.5%]
**Emo. input FT Model**	71.98%* [69.83%, 74.13%]	30.09%* [25.32%, 34.85%]	14.43%* [10.28%, 18.58%]	28.55%* [23.83%, 33.26%]	87.5%* [79.5%, 95.5%]

The test results of the original model and the fine-tuned model for each different optimization path. The results of each indicator are presented in the form of average values and 95% confidence intervals (in brackets).

* Denotes statistical significance (p-value<0.05) compared to the un-tuned model.

The experimental results show that the behavior-predicting LLM optimization approach can effectively improve its accuracy in predicting personnel behavior in high-pressure scenarios, with a satisfaction score of 67.5% and a satisfaction increase of 10 percentage points, a BERTScore increase of 3.57 percentage points, indicating that LLMs can learn the intrinsic patterns of homogeneous cultural behavior to some extent through fine-tuning. The emotion-predicting + behavior-predicting LLM optimization approach achieved a satisfaction score of 75.0%, representing an increase of 12.5 and 7.5 percentage points compared to the baseline model and the behavior-predicting LLM, respectively. Both BERTScore and ROUGE series metrics also improved, with BERTScore increasing by 4.92 and 1.66 percentage points respectively, indicating that LLMs can further improve their accuracy in predicting personnel behavior in high-pressure scenarios by predicting the immediate emotions of individuals. After implementing the emotion-input combined with behavior-predicting LLM optimization approach, the model’s behavior prediction capability showed clear improvement, achieving a satisfaction score of 87.5%. This represents an increase of 25 percentage points over the baseline model and a 12.5 percentage-point gain compared to the emotion-predicting + behavior-predicting LLM. Clear progress was also observed in BERTScore and ROUGE series metrics, with BERTScore increasing by 7.93 and 3.01 percentage points respectively.

We conducted paired t-tests for each metric of each fine-tuned LLM and their base LLMs. Apart from the ROUGE-2 metric of the “w/o Emo. FT Model”, all comparisons showed statistical significance (p < 0.05). Furthermore, we also conducted paired t-tests for each metric of the “FT Model” and the “w/o Emo. FT Model,” and the comparison showed statistical significance (p < 0.05). Exact p-values and mean differences along with their 95% confidence intervals, are presented in Table 4 in the S1 Appendix. Multiple comparisons are present, so the significance levels should be interpreted with caution.

This further indicates that LLMs can learn the intrinsic patterns of homogeneous cultural behavior to some extent through fine-tuning and verifies that the behavioral decisions of individuals are jointly driven by scenarios and emotions. This enlightens us that if we want LLMs to predict the behavioral decisions of individuals more accurately, we not only need to provide the LLM with immediate scenario information but also ideally provide the immediate emotions of the individuals. The emotion-predicting + behavior-predicting LLM optimization approach, through fine-tuning, enables the LLM to improve the accuracy of behavior prediction by predicting the immediate emotions of humans in situations where only scenario information is known and human immediate emotions cannot be known.

## Discussion

We utilized a dataset that exclusively describes U.S. law enforcement and security agencies to ensure cultural homogeneity and control for variability in emotional and behavioral responses. While this approach effectively mitigates differences stemming from national culture, it inadvertently emphasizes a relatively coherent institutional sub-culture that may not accurately represent broader human patterns of behavior.

The institutional sub-cultural norms inherent within these departments shape the emotional and behavioral responses of personnel. These norms dictate acceptable conduct, perceptions of stress, and emotional expressions in high-pressure situations, which may diverge from general civilian behaviors. Consequently, our model may be learning behavioral patterns that are specific to this institutional framework rather than universally applicable human behaviors. While we can conclude that our LLM enhances behavior prediction within the context of U.S. law and security enforcement agencies, applying these models to diverse environments (such as civilian emergency evacuation, Russian law enforcement actions, or other low-pressure environments) may not yield similar results. Future research should aim to incorporate a more varied dataset that includes multiple organizational cultures to better capture the complexities of human behavior in different contexts.

We reinforce the importance of careful consideration regarding the contexts in which AI predictions are deployed. The applicability of our findings should thus be explored cautiously, with an emphasis on contextual relevance and the potential influence of varied cultural and organizational norms on the emotional and behavioral dynamics at play.

While our study emphasizes the importance of anonymizing data to protect individual identities and maintain confidentiality, the broader ethical implications and AI safety concerns associated with developing such systems for high-stakes scenarios are also worthy of discussion.

The potential application of such technology in operational planning or assessments poses significant challenges. Predictive models could inadvertently reinforce existing biases, leading to bias amplification in decision-making processes. This risk is particularly pronounced in high-stakes environments, where misinterpretations of behavioral predictions may influence critical actions.

Furthermore, the automation of behavior predictions raises questions of accountability. Who is responsible when a predictive system leads to erroneous or harmful outcomes? The opacity of AI decision-making processes can hinder accountability, making it difficult to trace back decisions to specific model predictions or data inputs.

To address these challenges, it is essential to implement robust ethical frameworks and regulatory guidelines that govern the use of AI in high-pressure contexts. This includes continuous monitoring for bias, fostering transparency in how models are developed and utilized, and ensuring that stakeholders are engaged in discussions about the implications of these technologies.

## Conclusion

This study aimed to explore whether LLMs can improve the accuracy of predicting personnel behavior in high-stakes scenarios by forecasting emotions. To achieve this, we developed a novel “scenario-emotion-behavior” dataset based on homogeneous cultural data, and we fine-tuned LLMs using this rich contextual information. Through systematic experiments and validation, we compared various optimization paths to assess the impact of emotion prediction on behavior prediction accuracy.

The key results demonstrate that LLMs fine-tuned to predict emotions outperformed, on multiple metrics, both baseline models and those focused solely on behavior prediction. Specifically, our findings indicate a clear improvement in prediction capabilities when emotional context was integrated into the model. This validates our hypothesis that LLMs can enhance the accuracy of predicting personnel behavior by predicting emotions in high-pressure scenarios

Overall, the objectives of the study have been met, leading to several major inferences. First, our results establish that LLMs can learn the intrinsic patterns of behavior within homogeneous cultural contexts through fine-tuning. Second, the critical role of emotional information in refining behavior prediction capabilities has been highlighted, emphasizing the importance of considering emotional responses in decision-making processes.

The implications of these findings extend to both artificial intelligence and behavioral sciences, suggesting a promising framework for future research. By integrating emotional data with behavioral predictions, we lay the groundwork for developing more sophisticated LLMs capable of improving outcomes in high-risk domains. This study not only contributes to the interdisciplinary integration of AI with cognitive science but also fosters a new paradigm for understanding the dynamics of human behavior in critical contexts.

## Supporting information

S1 Appendix Tables 3 and 4. (DOCX)
