# Supplementary material for: Can LLMs improve the accuracy of behavior prediction for personnel in high-stakes scenarios by predicting emotions? — A fine-tuning study based on scarce homogeneous cultural data in high-pressure environments
Source: PLoS One. 2026 Jul 8;21(7):e0352988. doi: 10.1371/journal.pone.0352988 (PMC13345396; doi:10.1371/journal.pone.0352988)
Supplement: S1 Appendix — (DOCX) [file pone.0352988.s001.docx]

**Table 3. P-values and Mean Difference + 95%CI of Table 1.**

| **Model** | **BERTScore** | **ROUGE-1** | **ROUGE-2** | **ROUGE-L** | **Satisfaction Score** |
| --- | --- | --- | --- | --- | --- |
| **ERNIE-4.0** | 0.6596±0.0415 | 0.1799±0.0925 | 0.0400±0.0736 | 0.1693±0.0826 | 0.625±0.050 |
| **+FT** | 0.7332±0.0830 | 0.3851±0.2105 | 0.1606±0.2223 | 0.3595±0.2041 | 0.750±0.058 |
| **p-value** | 0.0000 | 0.0000 | 0.0000 | 0.0000 | 0.0077 |
| **Difference** | 0.0736  [0.0552, 0.0920] | 0.2052  [0.1596, 0.2508] | 0.1206  [0.0741, 0.1671] | 0.1902  [0.1465, 0.2339] | 0.1250  [0.1098, 0.1402] |
| **Llama-3.1** | 0.6363±0.0391 | 0.1124±0.0734 | 0.0227±0.0414 | 0.0931±0.0624 | 0.550±0.058 |
| **+FT** | 0.7301±0.0638 | 0.3821±0.1767 | 0.1665±0.2392 | 0.3682±0.1742 | 0.800±0.000 |
| **p-value** | 0.0000 | 0.0000 | 0.0000 | 0.0000 | 0.0016 |
| **Difference** | 0.0938  [0.0790, 0.1087] | 0.2697  [0.2317, 0.3077] | 0.1438  [0.0956, 0.1920] | 0.2751  [0.2384, 0.3118] | 0.2500  [0.2385, 0.2615] |
| **ChatGLM3** | 0.6169±0.0351 | 0.0816±0.0525 | 0.0087±0.0291 | 0.0680±0.0445 | 0.625±0.050 |
| **+FT** | 0.6443±0.0456 | 0.1384±0.0674 | 0.0230±0.0366 | 0.1261±0.0613 | 0.725±0.050 |
| **p-value** | 0.0000 | 0.0000 | 0.0011 | 0.0000 | 0.000000 |
| **Difference** | 0.0274  [0.0160, 0.0388] | 0.0568  [0.0399, 0.0738] | 0.0143  [0.0050, 0.0236] | 0.0581  [0.0431, 0.0731] | 0.1000  [0.0860, 0.1140] |
| **GPT-3.5** | 0.6751±0.0407 | 0.1613±0.0780 | 0.0613±0.1397 | 0.1444±0.0675 | 0.475±0.050 |
| **+FT** | 0.6948±0.0742 | 0.2669±0.1508 | 0.1059±0.1364 | 0.2560±0.1459 | 0.625±0.050 |
| **p-value** | 0.0036 | 0.0000 | 0.0034 | 0.0000 | 0.0069 |
| **Difference** | 0.0197  [0.0029, 0.0365] | 0.1056  [0.0719, 0.1393] | 0.0446  [0.0059, 0.0833] | 0.1116  [0.0797, 0.1435] | 0.1500  [0.1360, 0.1640] |
| **Qwen2.5** | 0.6215±0.0374 | 0.1011±0.0462 | 0.0101±0.0283 | 0.0892±0.0385 | 0.600±0.000 |
| **+FT** | 0.7356±0.0700 | 0.3846±0.2097 | 0.1961±0.2800 | 0.3631±0.2012 | 0.725±0.050 |
| **p-value** | 0.0000 | 0.0000 | 0.0000 | 0.0000 | 0.0077 |
| **Difference** | 0.1144  [0.0984, 0.1299] | 0.2835  [0.2409, 0.3261] | 0.1860  [0.1302, 0.2418] | 0.2739  [0.2333, 0.3146] | 0.1250  [0.1151, 0.1349] |

**Table 4. P-values and Mean Difference + 95%CI of Table 2.**

| **Configuration** | **BERTScore** | **ROUGE-1** | **ROUGE-2** | **ROUGE-L** | **Satisfaction Score** |
| --- | --- | --- | --- | --- | --- |
| **Base Model** | 0.6405±0.0537 | 0.1226±0.1140 | 0.0306±0.0652 | 0.1131±0.1026 | 0.625±0.050 |
| **+FT** | 0.6897±0.1042 | 0.2443±0.2578 | 0.0618±0.1187 | 0.2098±0.2273 | 0.750±0.058 |
| **p-value** | 0.0000 | 0.0000 | 0.0114 | 0.0000 | 0.0077 |
| **Difference** | 0.0492  [0.0262, 0.0722] | 0.1217  [0.0664, 0.1770] | 0.0312  [0.0047, 0.0577] | 0.0967  [0.0478, 0.1456] | 0.1250  [0.1100, 0.1400] |
| **w/o Emo. Model** | 0.6374±0.0515 | 0.1145±0.1043 | 0.0137±0.0419 | 0.0897±0.0778 | 0.575±0.050 |
| **+FT** | 0.6731±0.0673 | 0.1897±0.1504 | 0.0095±0.0167 | 0.1613±0.1303 | 0.675±0.050 |
| **p-value** | 0.0000 | 0.0000 | 0.8504 | 0.0000 | 0.0000 |
| **Difference** | 0.0357  [0.0191, 0.0523] | 0.0752  [0.0393, 0.1111] | -0.0042  [-0.0130, 0.0046] | 0.0716  [0.0419, 0.1014] | 0.1000  [0.0861, 0.1139] |
| **Emo. input Model** | 0.6576±0.0526 | 0.1834±0.1608 | 0.0393±0.1188 | 0.1414±0.1187 | 0.625±0.050 |
| **+FT** | 0.7198±0.1083 | 0.3009±0.2402 | 0.1443±0.2092 | 0.2855±0.2375 | 0.875±0.050 |
| **p-value** | 0.0000 | 0.0000 | 0.0000 | 0.0000 | 0.0016 |
| **Difference** | 0.0622  [0.0386, 0.0858] | 0.1175  [0.0608, 0.1742] | 0.1050  [0.0578, 0.1522] | 0.1441  [0.0921, 0.1961] | 0.2500  [0.2361, 0.2639] |
